# Complex Transition to Cooperative Behavior in a Structured Population Model

**DOI:** 10.1371/journal.pone.0039188

**Published:** 2012-06-25

**Authors:** Luciano Miranda, Adauto J. F. de Souza, Fernando F. Ferreira, Paulo R. A. Campos

**Affiliations:** 1 Departamento de Física, Universidade Federal Rural de Pernambuco, Recife-PE, Brazil; 2 GRIFE – Escola de Artes, Ciências e Humanidades, Universidade de São Paulo, São Paulo, Brazil; 3 Departamento de Física, Universidade Federal de Pernambuco, Recife-PE, Brazil; Hungarian Academy of Sciences, Hungary

## Abstract

Cooperation plays an important role in the evolution of species and human societies. The understanding of the emergence and persistence of cooperation in those systems is a fascinating and fundamental question. Many mechanisms were extensively studied and proposed as supporting cooperation. The current work addresses the role of migration for the maintenance of cooperation in structured populations. This problem is investigated in an evolutionary perspective through the prisoner's dilemma game paradigm. It is found that migration and structure play an essential role in the evolution of the cooperative behavior. The possible outcomes of the model are extinction of the entire population, dominance of the cooperative strategy and coexistence between cooperators and defectors. The coexistence phase is obtained in the range of large migration rates. It is also verified the existence of a critical level of structuring beyond that cooperation is always likely. In resume, we conclude that the increase in the number of demes as well as in the migration rate favor the fixation of the cooperative behavior.

## Introduction

The emergence of cooperation has puzzled philosophers and researchers since a long time ago. For instance, already in the seventeenth century Thomas Hobbes (1651) claimed that all people are egoists, they are primarily concerned with their own well-being, and act towards the defense of their own interests and properties [1]. Thus, the requirement for the cooperation among selfish individuals is the existence of a central authority. Nevertheless, cooperative behavior is ubiquitous in nature. One may find many examples in ecological and social sciences where cooperation exists in a decentralized way. Perhaps the greatest step to clarify this issue was given by Axelrod [2], [3]. Axelrod has surveyed the conditions under which the cooperative behavior may emerge in groups wherever the presence of a central force is lacking. His main finding was that the cooperation could emerge spontaneously between individuals who seek their own interests provided that a principle of reciprocity prevails among them.

The main legacy of Axelrod was to formalize the conflict and cooperation among selfish individuals in terms of the game theory approach, that has been demonstrated to be very appropriate to address this class of problem [4], [5]. The prisoner's dilemma Game (PDG) fits well in this category [6]. The PDG is a two-players game where at every encounter each player has to decide either to cooperate (C) or to defect (D). Whether one supposes that the individual aims to maximize his own gain rather than contributing to the collective benefit, then it is expected that in the prisoner's dilemma game betraying is the rational choice. Notwithstanding, this behavior does not hold when the game is iterated many times. Axelrod showed that in the game with repetition, in which the individuals are randomly matched, the cooperative behavior may coexist with the selfish behavior, a situation that remains stable throughout the evolution.

Later on, it was shown that the interplay between interaction rules and spatial structure can be an effective mean to promote cooperation [7]. The mechanism underneath the enhancement of the cooperation in spatially structured population is the cluster formation. The aggregation of cooperators enables them to get protected against the invasion of free riders [8]. This intriguing result has motivated many other investigations to depict the role of network topologies on the patterns of the cooperation among individuals. Within the framework of game theory, several topologies such as regular lattices [9], [10], regular random [11], random graphs [12]–[16] and complex networks [17]–[20] have been extensively studied and discussed in the literature [6]. These networks display a varying degree on the level of heterogeneity of connectivities, which increases as one changes from regular to complex networks. The heterogeneity of the interaction networks has been established as an important feature aiding the evolution of cooperation [15], [21]–[23], because cooperators profit from the occupation of densely connected nodes which by its turn help their dissemination. The identification of other mechanisms that lead to cooperation persistence on evolutionary PDG has been reported in the literature. Nowak highlighted the kin selection, directed reciprocity, indirected reciprocity, network reciprocity and group selection as the key rules [24].

Recently much attention has been paid to the role of mobility in different versions of the spatial prisoner's dilemma game. It is now recognized that migration can also be a very important mechanism influencing the fate of the cooperative behavior. Vainstein et al. studied the role of migration in systems with a fixed density of cooperators which are randomly placed in a two-dimensional square lattice [25]. According to their model, the agents look around in the nearest neighborhood to reproduce and migrate. They are allowed to copy the strategy of the individual with the highest payoff among their nearest neighbors (reproduction), and they are also allowed to move into an empty site with probability 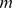 (migration). The reverse order of these actions produces distinct results. Their model exhibits interesting outcomes. In the former rule, where the copying process is followed by the migration step, it is observed the existence of a threshold for the migration probability 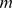, where beyond that point cooperators and defectors coexist. Below the threshold the cooperative behavior reaches extinction. When the sequence of these processes is reversed the meaning of the migration threshold alters. Now, migration values larger than the threshold implies the elimination of the cooperators. Vainstein et al. also verified that the density of individuals affects the fate of the cooperation. They ascertained that at low density the mobility has a detrimental effect and thereby destroys cooperation, whereas at high density the opposite happens and the mobility enhances cooperation. In order to prevent the exploitation by defectors, the cooperators should be disposed in compact clusters.

Following the ideas of Vainstein et al. [25] several other models highlighting the role of migration have been proposed [26]–[32]. From these investigations important new concepts have arisen such as the success-driven migration [26], [27], adaptive migration [28] and aspiration-induced migration [30], [31].

The current work inquires the role of migration in the onset and fixation of the cooperative behavior in subdivided populations. The study of the interplay between evolutionary mechanisms and the underlying topology where populations reside is an issue of great interest since a long time ago. The first steps in this direction was given by Kimura to study the problem of local differentiation on genes frequencies in a structured population model [33]. The model, known as the stepping-stone model, considers the population to be subdivided into colonies where in each generation individuals can migrate to nearby colonies. In standard representations, these subpopulations are arranged on a two-dimensional grid, such that the number of neighbors of each subpopulation is the same when periodic boundary conditions are assumed. Nowadays, it is well established that population structure plays a prominent role in adaptive evolution [34], [35], epidemiology [36], [37] and ecology [38], to cite just a few.

Likewise, our model assumes that the population comprises several smaller subpopulations (demes) which are randomly connected. These connections represent possible pathways where the individuals can move between neighbor demes. We study the prisoner's dilemma game in an evolutionary perspective. The evolutionary dynamics combines the game itself, adaptation and migration. As expected, migration plays an important role in driving the fate of the cooperative behavior. All the possible outcomes, namely, extinction of the population, dominance of the cooperative behavior and coexistence between cooperators and defectors, are found. The extent of these distinct regimes is greatly influenced by the migration rate and the level of structuring of the population (number of demes). In particular, it is found that an increased number of demes as well as an increased migration rate favor the fixation of the cooperation. However, in the regime of extremely large migration rates, where the coexistence between cooperators and defectors is assured, the level of cooperation diminishes as the exchange of migrants between the demes becomes more intense. Another important feature is the observation, in the range of low migration rates, of a critical level of structuring such that beyond that value cooperation can emerge.

In the next Section we describe the model. In the following we present the simulation results and discussions. At the end of the manuscript we present our conclusions.

## Methods

### The model

**Figure 1:**
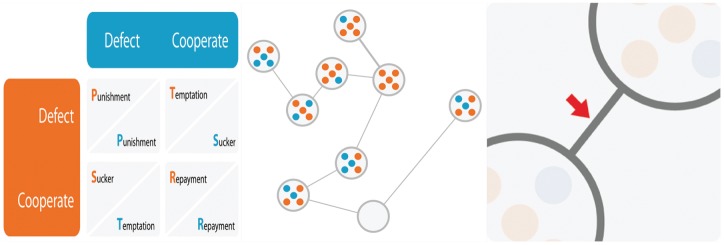
Description of the model. Left panel: The payoff matrix which describes the payoff value after one round of the prisoner's dilemma game between two players (blue and orange) according to their strategies. Middle panel: An illustration of the structured population model. In this instance the population comprises 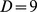 demes each one with carrying capacity 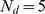. At this moment one of the demes is empty but recolonization is possible through migration from the two neighbor demes. Just to point out, after migration the number of individuals can exceed or be under the carrying capacity 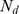. Right panel: The links represent possible paths where migrants can go through.

The population consists of 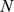 players which are distributed over 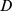 demes (subpopulations), such that initially each deme has initially 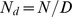 individuals. Each individual can adopt one of the two pure strategies: cooperate (C) or defect (D) (See Figure 1). The individuals interact and compete locally. The subpopulations interact through the exchange of migrants.

The population evolves on time according to the following life cycle: prisoner's dilemma game, migration and selection. During the prisoner's dilemma stage, in each deme one randomly constitutes 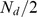 pairs of individuals which are then forced to play one round of the prisoner's dilemma game (PDG). At each round, individuals are rewarded according to their strategy and the strategy of their opponents. Following the original PDG, whenever a cooperator matches a defector, the defector receives the highest payoff T (temptation) while the cooperation is punished with the lowest payoff S (sucker). Whenever a cooperator matches another cooperator, both players get payoff R, whereas the encounter of two defectors means that both are compensated with payoff P. The payoffs satisfy the following condition 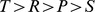. The additional requirement 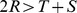 is needed in order to provide a non-null chance of the cooperative strategy to persist.

As already mentioned, the demes interact through migration. Every generation, each individual has a probability 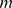 of migrating to one of the demes which is in its immediate neighborhood. After migration the size of each deme is not necessarily equal to 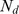. Subsequently selection works. Selection is local, i.e., it occurs isolatedly in each deme and consists in two steps. Firstly, those individuals whose fitnesses are below the elimination threshold, hereafter denoted by 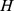, are automatically extinct. The elimination mechanism proposed by Zhang et al. [23] has been demonstrated to be an important feature in fostering the cooperation levels on evolving networks. The fitness depends on the individual's payoff and also on the fitness value in the previous generation according to [23].
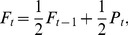
(1)where 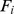 denotes the individual's fitness in generation 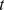, while 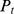 is the individual's reward at the game stage (payoff). The use of the generation-accumulated payoff as given by Eq. (1) embeds a mechanism of learning, which is strongly supported by studies in animal behavior [39], [40] and economics [41]. Note that in the way it is estimated, the fitness is bounded, and its maximum value is 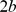. After the elimination step, three configurations are possible:

1. The simplest one corresponds to the situation where the size of the deme is exactly equal to 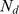. In this case nothing else happens.
2. The size of the deme is greater than 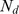, what is possible due to migration. In this case, an additional elimination process takes place until the group size is restored to its carrying capacity 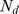. To proceed, the individuals are sorted according to their fitness: the least adapted player is eliminated, and the process is repeated until the carrying capacity is achieved.
3. The size of the deme is smaller than 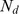. In this situation the population experiences a local expansion and again the deme size is restored to 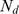. To proceed with the expansion individuals replicate with probability proportional to their fitness, i.e, the most adapted individuals have a greater chance of producing offsprings. In the replication, the daughter inherits the strategy of its parent, but not the fitness.

Figure 1 illustrates an instance of a structured population with 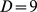 demes. In the example, the carrying capacity of each deme is 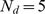. Cooperators and defectors coexist and one of the demes is empty. The deme can only be recolonized through migration of players from its neighborhood. It is important to stress that after migration the deme size is not necessarily equal to 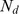, as already explained. When an individual migrates its fitness is not altered. This assumption is not restrictive. We have checked that whether one assumes that the evolutionary history of migrants is erased the results do not alter qualitatively.

As the topology for the migratory network we consider the random graph by Erdös and Rényi [42]. Accordingly, each pair of nodes of the graph is connected with probability 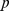 and thereby the total number of links is 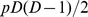. Thus, the mean connectivity of the graph (mean coordination number) is simply given by 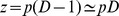. In the limit of large 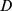, the distribution of connectivities follows a Poisson distribution like
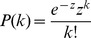
(2)


which is entirely described by the single parameter 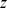.

## Results

During simulations we easily assess the concentrations of cooperators and defectors over time. Henceforth, the simulation data corresponds to the measurements carried out in the stationary regime, which by definition is characterized either by a fixed value of a given observable or when its value over time does not change within a given accuracy. The states 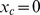 and 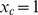, where 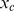 denotes the frequency of cooperators, are absorbing states, once cooperators or defectors are eliminated the evolutionary rule does not restore them. The key ingredient of the current model is structuring. Migration plays a crucial role because it can handle the degree of isolation of the groups. When the migration rate 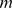 goes to zero, the patches behave independently, whereas when 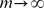 migration has a high homogenizing effect and the population behavior approaches that of a panmitic population (homogeneous population).

**Figure 2:**
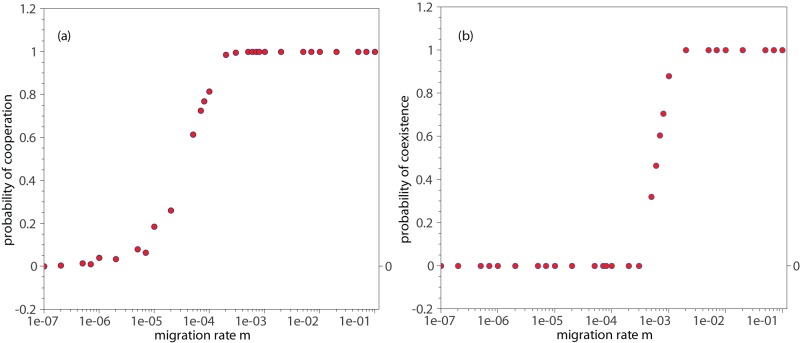
Evolution of the cooperative behavior with migration. Panel(a) Persistence of the cooperative behavior. The likelihood of fixation of cooperators as a function of the migration rate 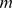. (b) Probability of coexistence between cooperators and defectors versus migration rate 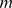. The parameter values are maximum population size 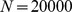, number of subpopulations 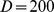, elimination threshold 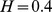 and payoff matrix elements 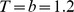, 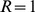, 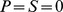. The data points are averages over 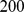 independent runs.

In Figure 2(a) we show the likelihood of fixation of the cooperative behavior. The fixation of the cooperation strategy occurs either when it becomes the dominant strategy, i.e. 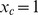, or when cooperators coexist with defectors in the population. To differentiate these situations Figure 2(b) displays the probability of coexistence between cooperators and defectors. For small enough migration rates, such that the level of isolation of the patches is high, the cooperative behavior is not sustainable even with the existence of the elimination mechanism which is expected to promote cooperation [23]. Under this situation, the population evolves to an all-defect state and posteriorly reaches extinction because a pure population of defectors can not persist (This situation is slightly distinct when the level of subdivision is pretty high (very large D), where there is a tiny chance of cooperation to persist, as we will see in the phase-diagrams). Around 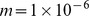 cooperators can thrive and the cooperative behavior can fixate in the population. At this point, one observes a non-coexistence regime and thereby either defectors or cooperators dominate the population. The probability of persistence of the cooperative behavior then rises with an increased migration rate up to finding ensured fixation around 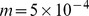. It is interesting to note that at this point the probability of coexistence becomes non-null and then starts to rise with migration. Coexistence is ensured at migration rate around 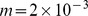.

According to the above scenario, without migration the dynamics leads the populations to extinction. As migration rate rises, the deme that had become empty due to the dominance of defectors can be recolonized by cooperators. These few newcomer cooperators then experience an immediate expansion and can achieve dominance. If the migration rate is further increased then the individuals are migrating continuously, and because the time scale of the movement throughout the population is short, defectors are constantly exploiting cooperators in favorable environments, where cooperators are the majority. When the defectors thrive and expand in a given deme, some of these cheaters can move to a new group before extinction of the entire deme. At the same time cooperators are more likely to migrate into the deme, and complete extinction of the local population is improbable. This is the regime where coexistence takes place.

**Figure 3:**
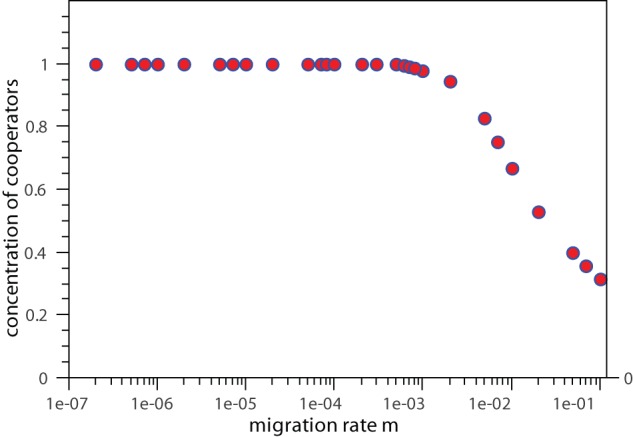
Cooperation level conditioned on the persistence of the cooperative behavior. Frequency of cooperators as a function of migration 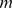. The parameter values are the same as in Figure 2. The data points are averages over 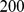 independent runs.

Figure 3 exhibits the mean concentration of cooperators conditioned on the fixation of the cooperative behavior. Observe that for low migration values where the maintenance of the cooperation is not very likely, the concentration of cooperators in the steady regime, conditioned on its persistence, is exactly equal to unity. This means that in this range of migration values cooperators can not coexist with defectors. As the migration rate rises the scenario remains the same up to migration rates of order 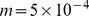 where coexistence becomes probable. Beyond this value the level of cooperation declines with 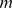. Though, the cooperation level is significantly larger than zero even for extremely high values of migration rates. In this phase, the mobility of the players is intense, and as pointed out before, coexistence between cooperators and defectors is ensured. The extinction of demes is also an unlikely event and now defectors can easily move to more favorable environments and increase their fitness values without compromising the survival of the cooperators that bear their existence. The higher the migration rate, the higher the concentration of defectors. In resume, a greater mobility requires a smaller frequency of cooperators to support the coexistence regime.

### Effect of population size and number of demes

**Figure 4:**
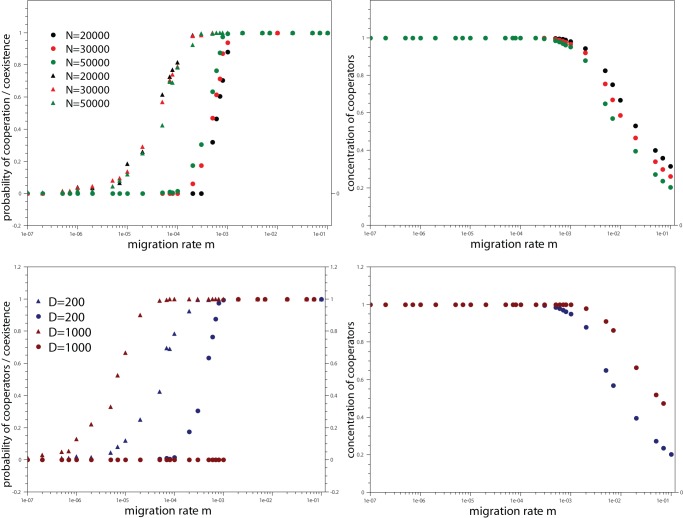
Effect of population size and number of demes on the outcomes of the model. Upper left panel: Probability of fixation of the cooperative strategy (triangles) and probability of coexistence between cooperators and defectors (circles) against migration rate 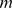 for distinct values of population size. Upper right panel: frequency of cooperators as a function of migration rate 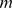 and different values of the population size and representation follows the one used in the left panel. In the upper panels the parameter values are 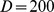, 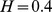, 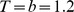, 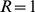, 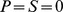. Lower left panel: Probability of fixation of the cooperative strategy (triangles) and probability of coexistence between cooperators and defectors (circles) against migration rate 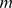 for fixed population size and different number of demes. Lower right panel: frequency of cooperators as a function of migration rate 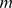 and different values of deme size and representation follows the one used in the left panel. The parameter values are 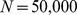, 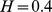, 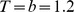, 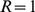, 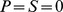. The data points are averages over 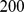 independent runs.

In the upper left panel of Figure 4 we examine the probability of fixation of the cooperative behavior and probability of coexistence between cooperators and defectors versus migration rate for different population sizes. When the number of demes is kept fixed and the population size is augmented, the carrying capacity 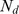 of the demes increases in the same way. Though, the population size has no major effect on the probability of fixation of the cooperative behavior, and the onset of the coexistence regime is slightly shifted to lower values of migration rates as 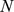 enlarges (upper right of Figure 4). A stronger effect of the population size is only seen in the frequency of cooperators. In the coexistence regime, the cooperation level clearly reduces with 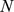.

In the lower panels we now address how the number of demes affects the results of the model. Now the population size is held constant, 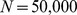, and two different values of the number of demes are simulated, 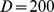 and 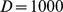. Major effects are observed, which proves that structure plays a crucial role in the fate of the cooperation. For larger 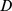, the onset of the cooperative regime shifts considerably towards a smaller migration rate. Instead, the opposite happens to the onset of the coexistence regime, which is then displaced to a larger migration rate. Additionally, the transition becomes sharper. These drifts in opposite ways now create a broad interval of migration values where the cooperative behavior is the only attractor of the dynamics, opposed to the verified for low 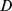 where the dominance of the cooperative behavior is constrained to a narrow region. One can readily infer from the lower left panel that for one decade of the migration rate 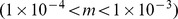 the cooperative behavior is the only possible outcome of the evolutionary process. Further, one can remark that the cooperation level in the coexistence regime is also affected, being considerably larger for a more subdivided population, as seen in the lower right panel.

**Figure 5:**
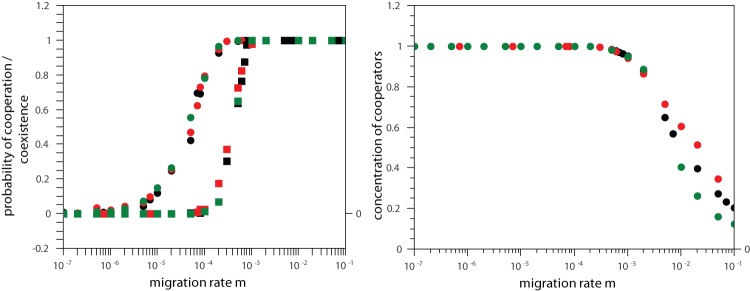
Dependence on the topology of the migration network. Left panel: Probability of fixation of the cooperative strategy (circles) and probability of coexistence between cooperators and defectors (squares) versus migration rate 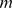. Right panel: Frequency of cooperators as a function of the migration rate 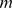. In both panels, the topologies of the migratory networks are: random graph (black symbol), scale-free networks (red symbols), and island model (green symbols). The parameter values are 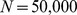, 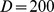, 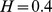, 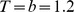, 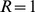, 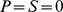.

**Figure 6:**
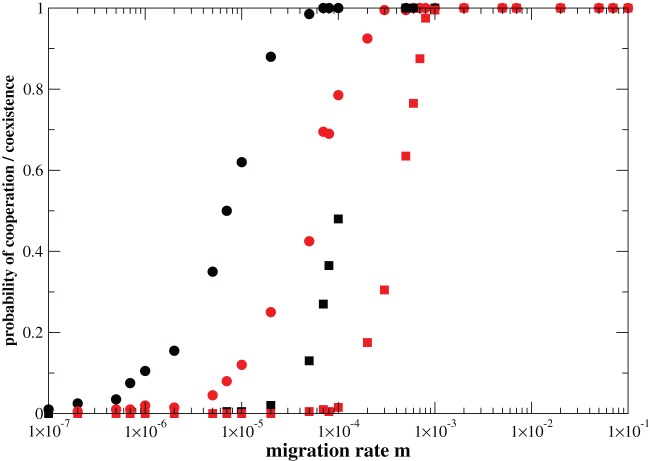
Effect of the elimination threshold 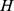 on the dynamics of the model. Probability of fixation of the cooperative strategy (circles) and probability of coexistence between cooperators and defectors (squares) versus migration rate 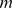. The parameter values are 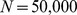, 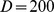, 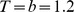, 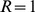, 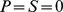. Black symbols correspond to elimination threshold 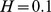, while red symbols denote 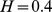.

The qualitative results of the model are very robust and poorly sensitive to the topology of the migration networks. To corroborate this ascertainment, Figure 5 left shows the probabilities of fixation of the cooperative behavior and coexistence for different topologies of the migratory network, namely, random graphs, island model, and scale-free networks. In scale-free networks the distribution of connectivities follows a power-law like 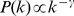 [43], [44]. In the island model, introduced by Wright [45], a single migration event is equally likely to move the individual to any other deme in the population, i.e., the connectivity of every deme is constant and equal to 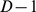. When looking at the probabilities of cooperation and coexistence, the results are nearly indistinguishable. Though, a consistent pattern of differentiation among the topologies arises when one analyzes the concentration of cooperators (Figure 5 right). In the regime of coexistence between cooperators and defectors, which corresponds to large values of migration rates, the larger the heterogeneity of the network, characterized by the variance of the connectivity distribution, the higher the sustained level of cooperation. In one extreme the island model has a null variance for the connectivity distribution and displays the smallest concentration of cooperators in the coexistence regime. On the other side, the scale-free networks has a diverging variance and sustain the highest levels of cooperation. The random graphs has a finite variance and fall in between these two extremes. The same happens to their sustained levels of cooperation. For the sake of completeness, in Figure 6 we study the role of the elimination threshold 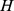 on the dynamics of the system. Once more, the results remain qualitatively unchanged. The most remarkable feature is the shift of the onset of the cooperative and coexistence regimes towards higher values of migration.

### Phase diagram

**Figure 7:**
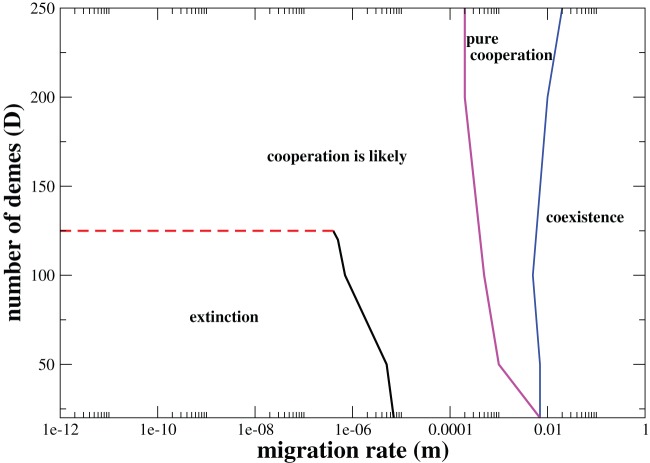
Phase diagram in the parameter space 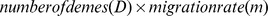. The parameter values are 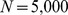, 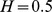, 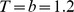, 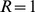, 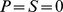. The red dashed-line denotes the critical value for the number of demes, 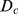, such that for any 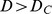 the chance of obtaining the cooperative behavior is greater than or equal to 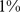.

**Figure 8:**
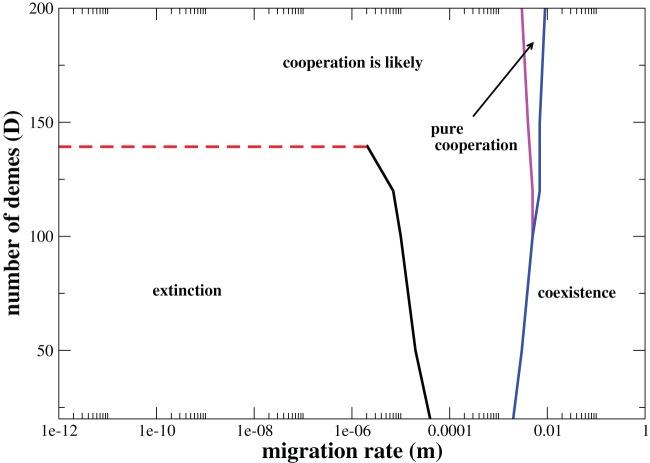
Phase diagram in the parameter space 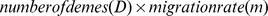. The parameter values are 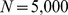, 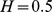, 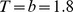, 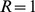, 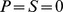. The red dashed-line denotes the critical value for the number of demes, 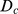, such that for any 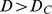 the chance of obtaining the cooperative behavior is greater than or equal to 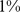.

To summarize the previous results Figures 7 and 8 sketch typical phase diagrams in the parameter space 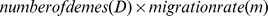. The lines delimit the four distinct regimes: (1) extinction, which occurs when defectors overcome cooperators and are subsequently extinct; (2) cooperation is likely, which denotes the region of the parameter space where the populations evolve either to a pure cooperative state with a non-null probability (but not equal to one) or goes to extinction; (3) the pure cooperation regime means that the only possible outcome is the dominance of the population by cooperators; and finally (4) coexistence phase, which denotes the region of stability where both cooperators and defectors can coexist. The outline of the phase diagrams is strongly influenced by the population size. In this way the population size is kept fixed, 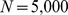 in both plots. One striking feature on the phase diagrams is the existence of a critical number of demes, denoted by 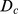. Above the threshold 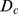 the likelihood that the system evolves to the cooperative behavior becomes non-negligible even when 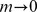. For the set of parameters of the Figure 7 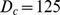, while in Figure 8 where the temptation payoff is larger 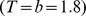 the threshold augments to 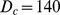. To delineate the lines we adopted as a criterion that the onset of cooperation corresponds to a probability of fixation which exceeds 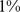. For a population of size 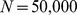, and assuming 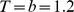, the critical level of structuring is almost ten-fold larger 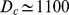 (data not shown). This result corroborates that finiteness can be very effective in shaping the evolution of the cooperative behavior. Because the fate in each deme is not deterministic, there is always a non-negligible likelihood that cooperation can fixate by chance in a given deme, which is larger as the strength of stochasticity becomes more intense, i.e., as smaller 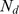 are simulated. When migration rate is too low, once cooperation fixates in a single or in a few demes, the remaining demes which are dominated by defectors will reach extinction. These empty demes can subsequently be recolonized by the successful cooperators. Of course, because 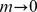, it will take a long time for the complete spreading of the cooperative behavior. A similar mechanism, known as multilevel selection, can promote cooperation on populations evolving on random networks [15], [16]. In that case, evolving random interaction networks can have an optimal response for the evolution of cooperation when the process of addition/deletions of links turns possible the formation of isolated homogeneous groups, which by its turn enhances the strength of the cooperators in the population [15], [16]. Actually, the term multilevel selection within the framework of the evolution of cooperation was first coined by Traulsen and Nowak [46]. Once more, it refers to the mechanism that promotes group selection where cooperators benefit from homogeneous groups while defectors do not.

As the migration rate is increased the extinction regime disappears and the regime where *cooperation is likely* becomes no more constrained to populations with number of demes larger than 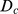. Sweeping the whole range of migration rates from low to large values one first observes that the system is propelled into a *pure cooperative* regime, where the dominance of the cooperative behavior is deterministic. The width of the pure cooperative regime grows with 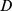. At this point, it is very important to point out the role of the temptation payoff 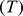. When the temptation payoff is high, it is possible for the system to change from the *cooperation is likely* phase to the coexistence phase without passing through the intermediate *pure cooperation* regime for small enough 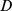 (see Figure 8).

For sufficiently large values of 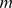, it is always possible to obtain the coexistence between the two strategies. A striking feature is that the coexistence regime signifies a double-edged sword for the evolution of cooperation: at the same time it warrants the fixation of the cooperative strategy, it also implies that a higher migration rate means that lower levels of cooperation are sustained.

## Discussion

This paper has investigated the conditions under which the cooperation is maintained in structured populations. The population comprises individuals that adopt pure strategies and have to decide to cooperate or to defect on every round of the prisoner's dilemma game. The problem is addressed in an evolutionary perspective, such that individuals which are most successful have a better chance of avoiding elimination and can contribute with more offspring. The main goal of the current work is to determine the importance of structuring in the evolutionary outcome and the required conditions for the sustaining of the cooperative behavior. The level of structuring is established by the number of subpopulations and the migration rate. When the number of demes is enlarged the population size within the demes becomes smaller, which increases the effects related to finiteness because reproduction is performed stochastically. The most strong evidence of the role of this stochastic force is the existence of a threshold for the number of demes beyond which there is a non-negligible chance of obtaining the fixation and dominance of the cooperative strategy even for extremely low values of migration. The importance of finiteness in the evolution of cooperation has already been demonstrated in the success rate of invasion of a single individual using the tit-for-tat strategy in a population of All-defect individuals [47].

Several recent investigations have addressed the role of mobility (migration) in spatially extended evolutionary games [25]–[27], [29], [48], [49]. Some of these works report the action of mobility in shaping spatiotemporal patterns that favor the robustness and maintenance of cooperation [26], [27], [48], [49]. Our simulation results demonstrate a paradoxical role of the migration mechanism in promoting cooperation in subdivided populations with no underlying spatial structure. At first glance, in the range at which migration rate is pretty small a gradual increasing of the migration rate has a beneficial effect in promoting the evolution of the cooperation. Low migration rates mean high level of isolation and thereby each subpopulation behaves as an independent population. Under this scenario, the selfish individuals defeat the cooperators and the population is subsequently extinct. Though, above a certain level of structuring 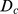, where stochastic events are even stronger, the elimination of cooperators by defectors is not mandatory and now cooperation is also likely to persist. As migration rate increases the extinction phase disappears, and now the system can evolve either to a cooperative or to a defective state no matter the number of demes. The existence of a pure cooperative behavior is also possible and verified for intermediate values of migration rates. Again, the level of structuring plays a prominent role in enhancing cooperation by increasing the width of the pure cooperative phase as 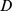 increases.

The detrimental effect of migration comes about in the range of large migration rates. Though elevated migration rates warrants the fixation of the cooperative strategy, it also enables the survival of defectors. Defectors survive by continuously exploring favorable subpopulations, which by turn are less prone to extinction because the high mobility of cooperators also enables them to more rapidly explore the demes which are facing degradation due to the growth of the local defector population. As 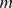 further increases a lower level of cooperation is needed in order to keep the coexistence between cooperators and defectors. In resume, higher levels of cooperation are obtained at intermediate values of migration rates.
